# Ultra-High-Performance Liquid Chromatography Tandem Mass Spectrometry Assay for Determination of Endogenous GHB and GHB-Glucuronide in Nails

**DOI:** 10.3390/molecules23102686

**Published:** 2018-10-18

**Authors:** Francesco Paolo Busardò, Massimo Gottardi, Anastasio Tini, Claudia Mortali, Raffaele Giorgetti, Simona Pichini

**Affiliations:** 1 Dep. of Excellence-Biomedical Sciences and Public Health, University “Politecnica delle Marche” of Ancona, 60020 Ancona, Italy; r.giorgetti@univpm.it; 2 Comedical S.r.L., 38100 Trento, Italy; Massimo.gottardi@comedical.biz; 3 Unit of Forensic Toxicology (UoFT), Department of Anatomical, Histological, Forensic and Orthopedic Sciences, Sapienza University of Rome, 00167 Rome, Italy; Anastasio.tini78@gmail.com; 4 National Centre on Addiction and Doping, Istituto Superiore di Sanità, 00167 Rome, Italy; claudia.mortali@iss.it (C.M.); simona.pichini@iss.it (S.P.)

**Keywords:** ultra-high-performance liquid chromatography tandem mass spectrometry, GHB, GHB glucuronide, nails, endogenous values

## Abstract

**Background**: The short chain fatty acid gamma-hydroxybutyric acid (GHB) is a precursor, and the metabolite of gamma-aminobutyric acid is commonly used as an illegal recreational drug of abuse. **Methods**: An ultra-high-performance liquid chromatography tandem mass spectrometry was developed and validated for endogenous GHB and its glucuronide in nails, to complement hair in forensic contexts for a retrospective detection of psychotropic drugs consumption. **Results**: GHB endogenous values for children and adolescents, adult females, and adult males in fingernails ranged from 0.3 to 3.0, 3.2, and 3.8 ng/mg, respectively, and toenails values ranged from 0.3 to 1.8, 2.0, and 2.4 ng/mg, respectively. In the three different groups, values of GHB in fingernails were statistically higher than those in toenails. GHB glucuronide could only be detected in finger nails with values ranging from 0.08 to 0.233, 0.252 and 0.243 in children and adolescents, adult females and adult males, respectively. **Conclusions**: The validated method was efficaciously applied to real finger and toe nails specimens from a population of males and females non GHB consumers. A preliminary cut-off of 5.0 ng/mg nail for endogenous GHB and 0.5 ng/mg for endogenous GHB-Gluc in the general population was proposed.

## 1. Introduction

The short chain fatty acid gamma-hydroxybutyric acid (GHB) is a precursor and metabolite of gamma-aminobutyric acid (GABA) and behaves as an inhibitory neurotransmitter in the central nervous system.

Its sodium salt, sodium oxybate, is approved as an adjuvant medication for detoxification and withdrawal of alcohol dependence (Alcover^®^) in some countries and for the treatment of narcolepsy-associated cataplexy (Xyrem^®^) [1].

Nevertheless, the most common use of GHB is as an illegal recreational “club” drug marketed for its ability to produce euphoria and sexual arousal [2]. In particular, this drug is gaining importance in combination with other psychoactive and non-psychoactive drugs such as mephedrone, methamphetamine, erectile dysfunction agents and alkyl nitrites (or poppers) in the context of “chemsex”: intentional or non-intentional intake of certain psychoactive and non-psychoactive drugs in the context of rave parties eventually followed by sexual encounters with the aim of aiding and/or enhancing the sexual relationship, mainly in homosexual settings [3,4].

The dual nature of endogenous neurotransmitter and exogenous pharmacologically active compound makes the proof of GHB intake a difficult assignment [5].

Cut-offs have been proposed for traditional biological matrices (e.g., blood and urine) to objectively discriminate exogenous drug consumption from endogenous values in antemortem and post-mortem samples [6]. However, in both biological fluids, GHB presents a short window of detection (around 5 h blood and less than 12 h urine) [7,8], so GHB and its glucuronide (GHB-Gluc) have been investigated in hair as potential biomarkers of GHB single and repeated intake [5]. For this purpose, baseline hair GHB values in the general population have been established to distinguish even a single intake, e.g., in GHB-facilitated sexual assaults [5]. However, the currently accepted approach to documenting a single GHB exposure in hair suggests “to use each subject as its own control” [5].

Nail testing can be accomplished to replace and/or complement hair testing in forensic contexts for the retrospective detection of psychotropic drug consumption. As the nail grows, ingested xenobiotics are incorporated into the keratin matrix, where they can be gathered for protracted periods of time (3–5 months in fingernails, and 8–14 months in toenails), allowing retrospective detection of drug consumption [9,10].

Considering that GHB has never been investigated in nails, we sought to develop and validate an easily applicable and fast ultra-high-performance liquid chromatography tandem mass spectrometry (UHPLC–MS/MS) method with rapid sample preparation for the determination of endogenous GHB and GHB-Gluc in nails. Secondly, finger and toe nails were compared to look for any eventual difference in GHB endogenous concentrations.

## 2. Results and Discussion

### 2.1. UHPLC–MS/MS and Validation Parameters

**Figure 1:**
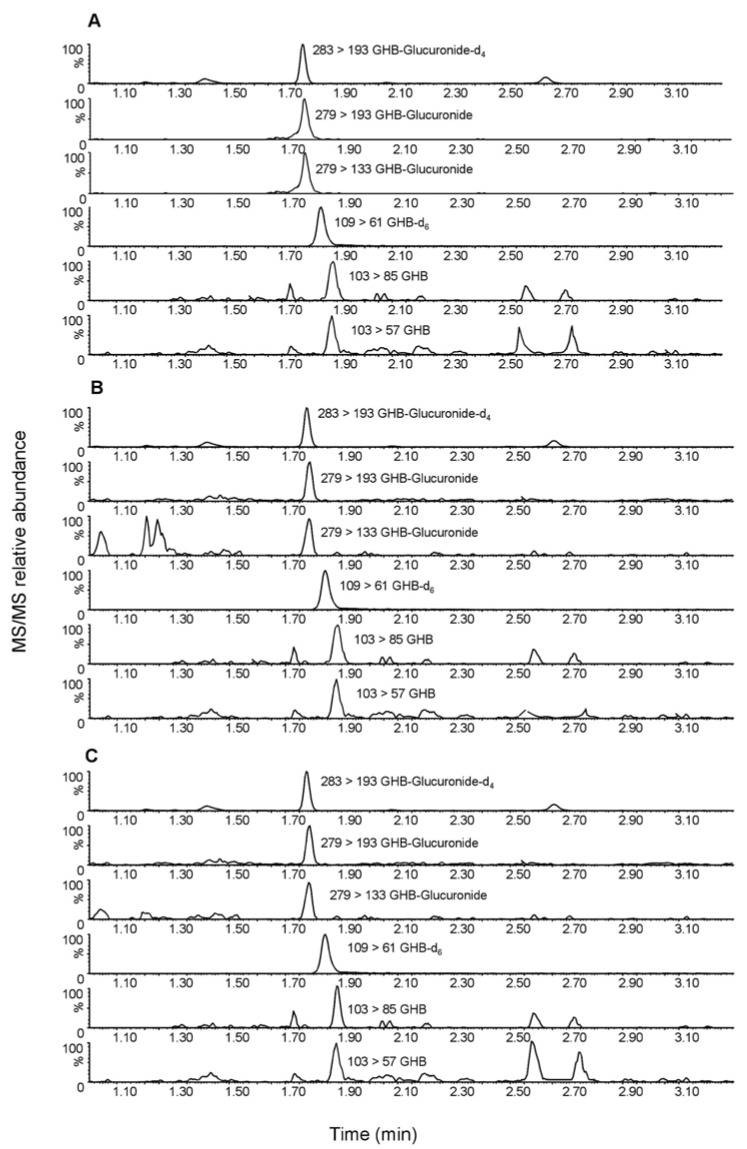
UHPLC–MS/MS chromatogram of an extract of (**A**) child finger nails containing 0.91 ng/mg GHB, 0.15 ng/mg GHB-gluc and 0.5 ng/mg ISs; (**B**) female finger nails containing 1.42 ng/mg GHB and 0.21 ng/mg GHB-gluc; (**C**) male fingernail containing 1.63 ng/mg GHB, 0.92 ng/mg GHB-gluc and 0.5 ng/mg ISs.

Representative chromatograms obtained following the extraction of GHB and GHB-gluc from child finger nails, female finger nails and male finger nails are shown in Figure 1A–C. Retention times of the two analytes were: 1.81 min for GHB-gluc and 1.87 min for GHB. A chromatographic run was completed in 10 min.

Determination coefficients (r^2^) of calibration curves were equal to or higher than 0.99 in all cases. Calculated LOD and LOQ values were suitable for the aim of the present study (Table 1). Precision and accuracy of LOQs always showed coefficients of variation lower than 20%. For all the other QC samples, the intra- and inter-assay precision and accuracy values met the internationally established acceptance criteria (Table 2) [11,12].

No significant analyte degradation was noted after one and three months storage of nails at room temperature, with differences being less than 10% from the initial GHB and GHB-Gluc concentration.

There was no carryover detected when injecting drug-free samples after the calibration curve’s highest point. No additional peaks from eventual endogenous substances from the keratin matrix were observed following the injection of drug-free nails. Similarly, none of the most common psychoactive drugs (cannabinoid, cocaine, opiates, amphetamines type-stimulants) or common psychoactive medications (e.g., benzodiazepines and antidepressants) interfered with the assay. No significant ion suppression (less than 10% analytical signal suppression) due to matrix effect occurred during chromatographic runs.

### 2.2. Analysis of Biological Samples

As reported above, the validated UHPLC–MS/MS method was used to measure endogenous values of GHB and GHB-Gluc in finger and toe nails collected from 30 children and adolescents, 30 adult females and 30 adult males (Table 3). From the obtained results, it can be said that in the case of GHB, similar values were measured for children and adolescents, adult females and adult males, with fingernail values ranging from 0.3 to 3.0, 3.2 and 3.8 ng/mg, respectively, and toenails values ranging from 0.3 to 1.8, 2.0 and 2.4 ng/mg, respectively. In the three different groups, values of GHB in fingernails were statistically higher than those in toenails. GHB glucuronide could only be detected in finger nails with values ranging from 0.08 to 0.233, 0.252 and 0.243 in children and adolescents, adult females and adult males, respectively. With these values available, we can propose a preliminary cut-off of 5.0 ng/mg nail for endogenous GHB and 0.5 ng/mg nail for GHB-gluc in the general population. This value necessarily has to be substantiated in a higher number of individuals with different age, gender and ethnicity.

## 3. Material and Methods

### 3.1. Chemicals and Materials

**Figure 2:**
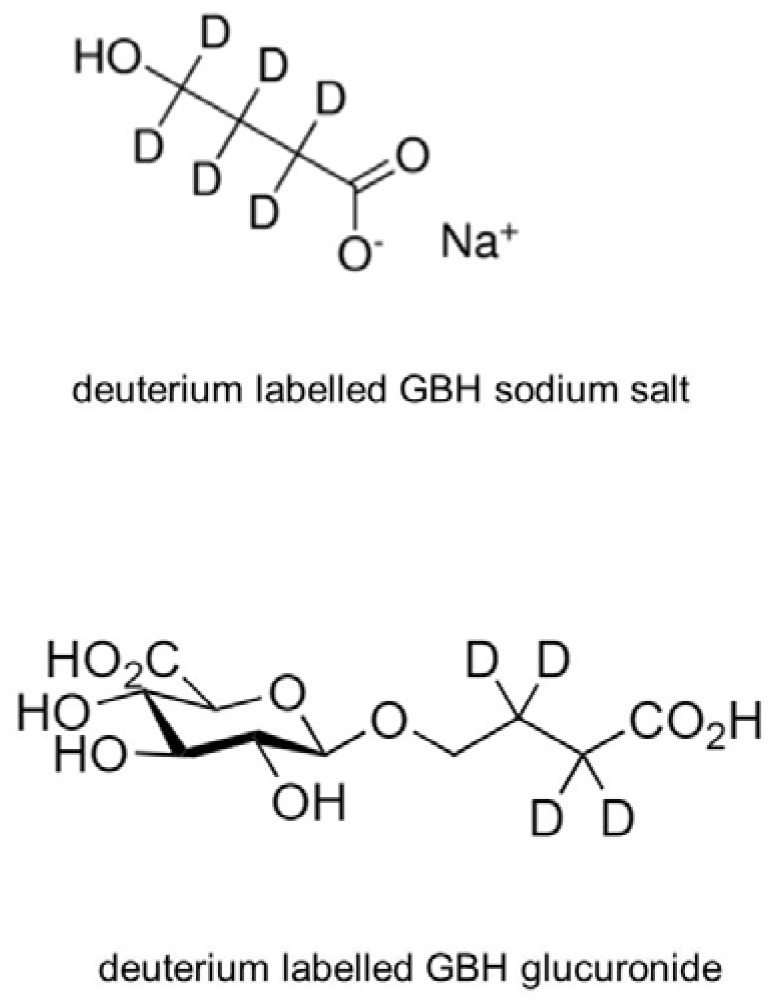
Deuterium labelled GBH sodium salt and deuterium-labeled GHB glucuronide.

GHB, pure standard (>99%) was purchased from Sigma-Aldrich (Milan, Italy). GHB-d_6_, used as internal standard (IS), was supplied as a methanolic solution by Sigma-Aldrich (Milan, Italy). Standards of the O-glucuronide derivative of GHB (GHB-Gluc) and its deuterium-labeled IS (GHB-Gluc-d_4_) were synthesized by Pedersen et al. [13] and provided by the Department of Drug Design and Pharmacology, Faculty of Health and Medical Sciences, University of Copenhagen (Prof. D.S. Pedersen). Deuterium-labeled GBH sodium salt and its glucuronide are reported in Figure 2.

VMA-TM3 (acidic aqueous buffer) reagent for nails digestion, diluent, washing solution and multimatrix eluent were obtained from Comedical s.r.l. (Trento, Italy). Oasis PRiME HLB solid phase extraction columns were from Waters, Milano, Italy. Ultrapure water and all other reagents of UHPLC–MS grade were acquired from Sigma-Aldrich (Milan, Italy).

### 3.2. Calibration Standards and Quality Control Samples

Methanolic standard solutions (10 and 1 mg/mL) and working solutions (10 and 1 μg/mL) of GHB and GHB-Gluc were stored at −20 °C.

Calibration standards of GHB and GHB-Gluc from limit of quantification (LOQ) to 10 ng GHB and 1 ng/mg GHB-Gluc per mg nails were prepared by daily spiking blank neonates nails to test linearity for each analytical batch.

Quality Control (QC) samples at three concentrations (low, medium, high) spanning the linear dynamic ranges of the calibration curves were also prepared by daily spiking blank nails with volumes of GHB and GHB-Gluc standard solutions appropriate to each analytical batch in order to check validation parameters (e.g., accuracy, precision, recovery, etc.) [11,12].

### 3.3. Biological Sample Collection and Preparation

GHB-free nails were generously donated by personnel of the research institutions participating in the study and by their relatives. Nail donors signed an informed consent for themselves and their children, when the latter also provided their nails. Since nails are waste material, spontaneously donated by participants, no ethical approval was required.

In detail, finger and toe nails were collected from 30 children and adolescents (range 5–16 years), 30 adult females (range 22–56 years) and 30 adult males (21–54 years). Nails were stored in paper envelopes at ambient temperatures until analysis. Nails were clipped as close to the nail bed as comfortable over a clean sheet of paper to collect the clippings, in order to obtain 2–3 mm of clippings from each of the 10 digits. Neonate nails, used as blank-free matrix, were donated by the Clinic Hospital of Barcelona as discharged material.

Nails were firstly washed with 2 mL dichloromethane and dried under nitrogen. A 25 mg sample was combined with 5 μL mixture of ISs (0.5 ng/mg), 0.5 mL Comedical VMA-TM3^®^ Reagent and kept at 100 °C for 1 h. Nails were then discarded, and the liquid mixture underwent solid phase extraction with Oasis PRiME HLB. Specifically, 0.5 mL mixture added to 0.5 mL Comedical Diluent^®^ was loaded in an HLB column, washed with 0.5 mL Comedical Washing Solution^®^, dried under nitrogen, and eluted with 0.5 mL Comedical Multimatrix Eluent^®^. The eluted mixture was diluted 1:10 with water and 1 μL injected into UHPLC-MS/MS system.

### 3.4. UHPLC–MS/MS

A Waters Acquity UPLC I-Class chromatograph (Waters, Milano, Italy) was used interfaced to a TQ-S micro triple quadrupole mass spectrometer (Waters). Analytes separation was achieved using an HSS T3 reversed phase C18 column (1.8 μm, 2.1 × 150 mm) from Waters.

A mixture of 95% solvent A (0.1% formic acid in water) and 5% solvent B (methanol) was used as mobile phase in isocratic conditions at a flow rate of 0.35 mL/min for 2 min. Then, the column was washed for 5 min, bringing solvent A to 5%, and then the mobile phase was re-equilibrated to initial conditions in 3 min.

**Figure 3:**
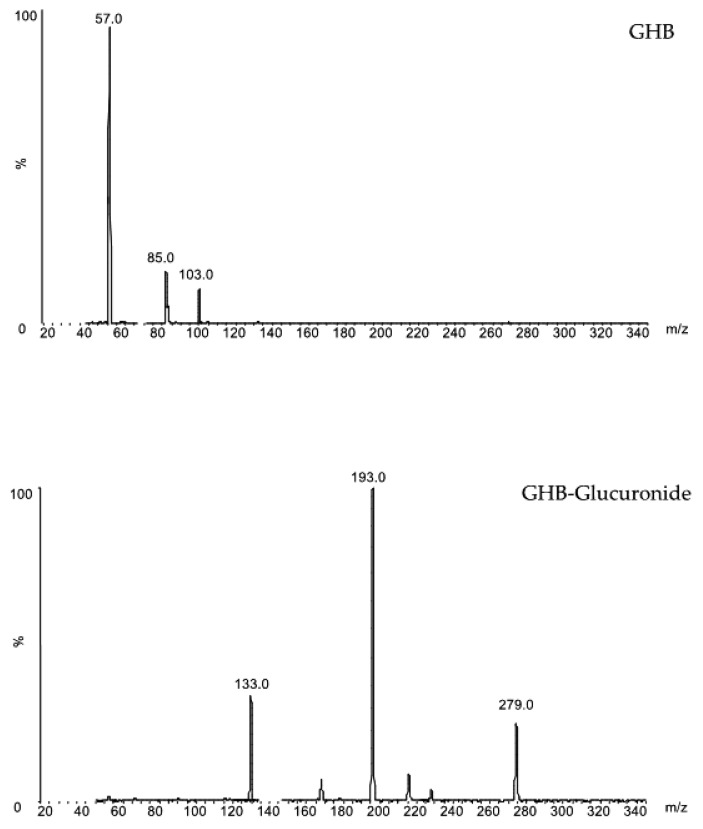
MS/MS spectra of GHB and GHB-Gluc.

A triple quadrupole mass spectrometer was used to detect analytes operating in multiple reaction monitoring (MRM) mode via negative electrospray ionization (ESI) The applied ESI conditions were as reported: capillary voltage −2.0 kV, desolvation temperature 650 °C, source temperature 150 °C, cone gas flow rate 20 L/h, desolvation gas flow rate 1200 L/h and collision gas flow rate 0.13 mL min^−1^. Cone voltage and collision energy were 20 V to 45 V. MRM transitions were the following: for GHB *m*/*z* 103 > 57 and *m*/*z* 103 > 85; for GHB-glucuronide *m*/*z* 279 > 193 and *m*/*z* 279 > 133; for GHB-d_6_ 109 > 61 and for GHB-Gluc-d_4_ 283 > 193. Transitions in bold were used for quantification. MS/MS spectra of GHB and GHB-Gluc are reported in Figure 3.

### 3.5. Validation of Analytical Method

The method developed following international criteria [11] was tested in a validation protocol following the most recent standard practices [12]. Linearity, limits of detection and quantification, accuracy, precision, recovery selectivity, carryover, stability and matrix effect were determined as previously described [14]. Five different daily replicates of calibration points and QC samples (low, medium, and high QCs) were used to calculate validation parameters along three successive working days. The above reported samples were obtained by spiking opportune concentrations of analytes under investigation and ISs in neonates nails, which were pre-analyzed and did not show measurable concentrations of GHB and GHB-Gluc.

## 4. Conclusions

We described a UHPLC–MS/MS assay which for the first time allowed the simultaneous identification and quantification of GHB and GHB-Glu in nails. The method was validated and applied to the determination of endogenous GHB and GHB-Gluc values in children and adolescent, adult women and adult males. For the first time, baseline values of GHB in nails have been preliminarily suggested, prompting investigation on a larger number of individuals from the general population of non GHB consumers.

## Figures and Tables

**Table 1:** Calibration method for GHB and GHB-Glucuronide in nails.

Analyte	Calibration Line		Determination Coefficient (r^2^)	LOD ^a^	LOQ ^a^
	Slope ^a^	Intercept ^a^		ng/mg	ng/mg
GHB	0.112 ± 0.0015	0.007 ± 0.023	0.997 ± 0.002	0.10	0.30
GHB-Glucuronide	0.171 ± 0.01	0.031 ± 0.04	0.996 ± 0.003	0.02	0.07

^a^ Mean and S.D. of five replicates on three subsequent working days.

**Table 2:** Intra-assay (n = 5) and inter-assay (n = 15) precision, accuracy (n = 15) and recovery of GHB and GHB-Glucuronide in nails.

Analyte	Intra-Assay Precision (% CV)			Inter-Assay Precision (% CV)			Accuracy (% Error)			Recovery (%)
	Low QC *	Medium QC *	High QC *	Low QC	Medium QC	High QC	Low QC	Medium QC	High QC	
GHB	2.4	2.3	2.0	3.6	4.1	2.0	5.0	3.8	2.5	96.5
GHB-Glucuronide	2.1	0.5	1.2	2.4	1.0	1.1	8.4	3.4	2.0	87.8

* Low QC: 1 ng/mg GHB and 0.1 ng/mg GHB-Glucuronide. Medium QC: 5 ng/mg GHB and 0.5 ng/mg GHB-Glucuronide. High QC: 8 ng/mg GHB and 0.8 ng/mg GHB-Glucuronide.

**Table 3:** GHB and GHB-gluc mean values in finger and toe nails collected from 30 children and adolescents, 30 adult females and 30 adult males.

	Children and Adolescents		Adult Females		Adult Males	
NAILS GHB (ng/mg nail)	Fingernails	Toenails	Fingernails	Toenails	Fingernails	Toenails
MIN	0.30	0.30	0.30	0.30	0.30	0.30
MAX	3.00	1.80	3.20	2.00	3.80	2.40
MEAN	1.51	0.91	1.62	0.96	1.78	1.13
SD	0.87	0.42	0.80	0.44	0.99	0.58
*p* value Fingernails vs. toenails		<0.01		<0.001		<0.01
NAILS GHB-gluc (ng/mg nail)						
MIN	0.082	ND	0.080	ND	0.082	ND
MAX	0.233	ND	0.252	ND	0.243	ND
MEAN	0.160	ND	0.153	ND	0.166	ND
SD	0.038	ND	0.048	ND	0.038	ND

MIN: minimum value. MAX: maximum value. SD: standard deviation. ND: not detected (under LOD value).
